# Electrocatalytic Reactions for Converting CO_2_ to Value‐Added Products

**DOI:** 10.1002/smsc.202100043

**Published:** 2021-08-08

**Authors:** Yueli Quan, Jiexin Zhu, Gengfeng Zheng

**Affiliations:** ^1^ Laboratory of Advanced Materials Department of Chemistry and Shanghai Key Laboratory of Molecular Catalysis and Innovative Materials Faculty of Chemistry and Materials Science Fudan University Shanghai 200438 China; ^2^ State Key Laboratory of Advanced Technology for Materials Synthesis and Processing Wuhan University of Technology Wuhan 430070 Hubei China

**Keywords:** coupled electrolysis, electrocarboxylation, electrocatalysts, electrochemical CO_2_ reduction, electrosynthesis

## Abstract

Converting CO_2_ into valuable chemical products has been intensively explored in recent years. Benefited from the substantial cost reduction of clean electricity, the electrochemical methods have been emerging as a potential means for CO_2_ conversion and fixation. Direct electrochemical CO_2_ reduction reaction (CO_2_RR) with H_2_O is achieved with continuously improved efficiency, selectivity and stability. In contrast, the coupled CO_2_RR with small molecules and organic substrates, which can allow to form higher valuable chemicals, is still hindered by the poor selectivity, unclear reaction mechanisms, and suboptimal performances of electrocatalysts. Herein, the development of CO_2_RR with electrocatalysts and reaction mechanisms is first introduced. Several representative examples are described for emphasizing concepts and methodologies. The research process and reaction mechanisms of the coupled CO_2_RR are then briefly discussed. Finally, challenges and perspectives in this field are addressed to further inspire the development of the fundamental understanding of reaction mechanisms for coupled CO_2_RR, as well as the optimization of electrocatalysts, electrolytes, and electrolyzers with high activity and selectivity.

## Graphical abstract

Electrochemical CO_2_ reduction reaction (CO_2_RR) toward high‐energy‐density chemicals/fuels offers a promising opportunity to achieve carbon neutralization. The reaction mechanism and electrocatalysts of CO_2_RR are intensively investigated, whereas the coupled CO_2_RR with small molecules and organic substrates are yet to be explored. Herein, the advances of electrocatalysts and reaction mechanism are summarized for the development of the coupled CO_2_RR.

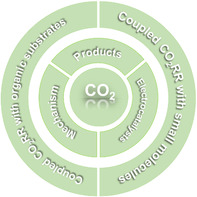

## 1 Introduction

**Figure 1:**
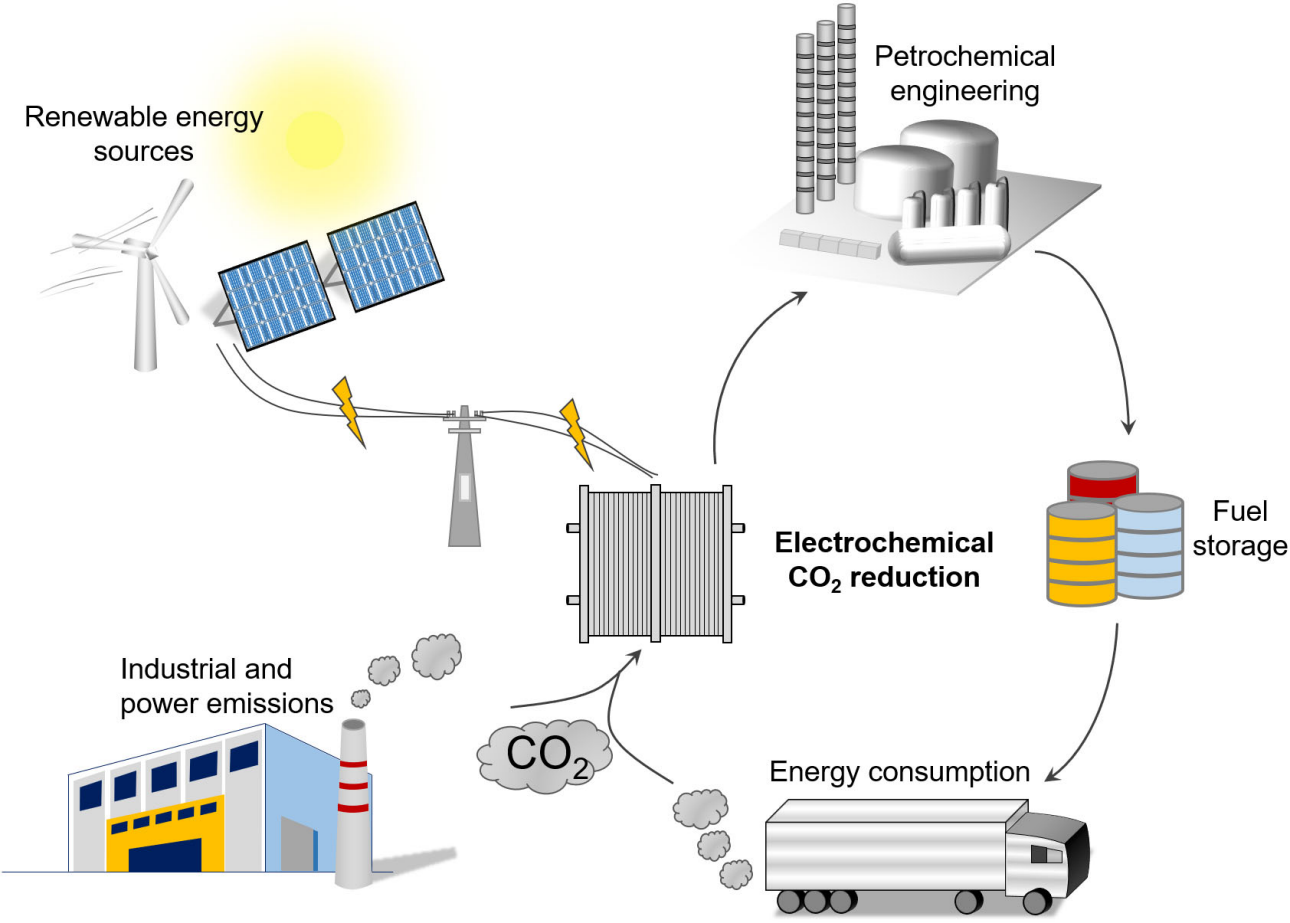
The schematic diagram of electrochemical conversion of CO_2_ into fuels and chemicals using renewably sourced electricity.

The sustainable developments of our society have been challenged by excessive consumption of fossil fuels and emissions of carbon dioxide (CO_2_), resulting in global warming and other serious environment hazards.^[^ 1, 2 ^]^ Over the past century, the industrial activities have consumed a huge amount of fossil fuels. Chemical products used today are obtained from fossil feedstocks by numerous processes in oil refineries, and most consumed fossil fuels are eventually converted into CO_2_. The electrochemical conversion of CO_2_ into high‐energy‐density chemicals and fuels offers a promising opportunity to neutralize these processes and reduce consumption of fossil fuels.^[^ 3, 4, 5 ^]^ With the development of renewable energy sources such as solar, wind, and tidal energies, the cost of renewable electricity has become competitive with electricity production from fossil fuels. It is ideal to use electricity generated by renewable energy to convert CO_2_ from industrial manufacturing and daily activities into value‐added chemicals/fuels, which can reduce emissions of CO_2_ and close the carbon loop (**Figure** **1**). The implementation of this loop can make a substantial effect on both the energy crisis and the greenhouse effect. Moreover, storing electricity in the form of chemical energy can effectively improve the utilization of excess electricity and produce more valuable products. For instance, ethylene is an important raw material to produce plastic, and ethanol can be used as fuels directly.^[^ 6, 7 ^]^ The energy density of propanol is higher than gasoline when used as fuels. Therefore, the electrocatalytic CO_2_ reduction reaction (CO_2_RR) and coupling with small molecules or organic substrates have been greatly intriguing chemists and materials scientists in the past couple of decades.^[^ 8, 9, 10, 11 ^]^

Direct CO_2_RR has several potential products such as syngas,^[^ ^12^ ^]^ methane,^[^ ^13^ ^]^ formic acid,^[^ 14, 15 ^]^ ethanol,^[^ ^16^ ^]^ and ethylene,^[^ ^17^ ^]^ which can be used directly as fuels or further to produce chemicals. Different products can be obtained via CO_2_RR depending on the catalysts used. Progresses to date have been able to achieve the production of molecules with carbon number less than 3 (i.e., C_1_, C_2_, and C_3_), whereas higher hydrocarbons and more complex chemicals are yet to be directly synthesized from electrochemical CO_2_RR. Although CO_2_RR is a promising way to achieve energy utilization, many challenges are still needed to be overcome, including the following. 1) The linear molecule CO_2_ is stable and hard to be activated. 2) The overpotentials for certain products are too high and consume too much energy. 3) The products have low selectivity and generally coexist, thus requiring additional separation processes. 4) Electrodes or catalysts have poor durability.^[^ ^18^ ^]^ Substantial efforts have been dedicating to the investigation of electrocatalysts, electrolytes, and electrolytic cells. Despite considerable advances achieved in recent years, it is still challenging to simultaneously achieve low overpotentials, industry‐level high current densities, and long durability for multicarbon (C_2+_) products.^[^ 1, 19 ^]^ Industry‐level current densities exceeding 0.5 A cm^−2^ with over 500 h of stability still remain a major target.^[^ 6, 20, 21 ^]^ In this regard, the development of CO_2_RR needs to be focused on pursuing highly efficient and robust materials with low energy barrier to activate CO_2_, adequate electrolytes, and stable operating devices.

Generally, multicarbon chemicals are more attractive due to their higher values and energy densities. For instance, it may become profitable to synthesize isopropanol through CO_2_RR with a current density over 300 mA cm^−2^, an overpotential less than 0.5 V, and a Faradaic efficiency (FE) higher than 70%. However, reducing CO_2_ into multicarbon suffers from low product selectivity and substantial energy cost.^[^ ^3^ ^]^ Thus, coupling intermediates obtained by CO_2_ reduction with small molecules and organic substrates has also attracted more attention from researchers, which may allow to obtain target products with higher economic benefits through electrocatalytic reactions.^[^ ^22^ ^]^ The small molecules and organic substrates reactants can be derived from anode products or electrolyte additives. High selectivity and activity toward C_1_ products have been widely reported in CO_2_RR.^[^ ^23^ ^]^ The generated *CO or *C═C═O is capable of bonding with other reactants like NH_3_ to produce acetamide, which is more economic and valuable.^[^ ^24^ ^]^ In addition, the activated CO_2_ (CO_2_^•−^) can directly react with organic substrates such as olefins, alkynes, and carbonyl compounds into the carboxylic acid compound, known as the electrocarboxylation reaction.^[^ 11, 25, 26 ^]^ Thus, it is economic and attractive to develop the coupled CO_2_RR with small molecules and organic substrates to obtain chemical raw materials. However, with the introduction of other reactants, four types of competing reactions can exist under the applied bias voltage, including hydrogen evolution reaction (HER), CO_2_RR, coupled CO_2_RR, and small molecules/organic substrates’ reduction reaction. Consequently, it is a much more complex reaction system than CO_2_RR. The high single‐product selectivity and partial current density are hard to be achieved. The electrolysis system, including reactants’ concentration, electrocatalysts, electrolytes, and electrolyzers, plays an important role, but among them the most key should be electrocatalysts for product selectivity. In CO_2_RR, the exploration of the reaction mechanism and the preparation of electrocatalysts with high selectivity and activity have been initially realized. Some optimization strategies are worth learning to achieve the high product selectivity of coupled electrolysis.

There are two ways to enable the coupled CO_2_RR. One is to reduce CO_2_ and small molecules/organic substrates in one electrolytic cell, and the other is to obtain the CO_2_ reduction product in one electrolytic cell and then deliver it into another electrolytic cell to achieve coupled electrolysis. Considering the complexity of CO_2_RR products, it is hard to transfer a single product to the second electrolytic cell, so that the product selectivity of coupled electrolysis can be more complicated. In addition, the concentrations of CO_2_RR products are typically quite low in electrolytes, which further reduces the reaction rate of coupled CO_2_RR. In contrast, when the CO_2_ reduction and coupling with small molecules/organic substrates take place in one single electrolytic cell, the reactant molecules can directly react with active CO_2_^•−^ on the electrocatalysts’ surface, greatly improving the reaction rates and product selectivity.

**Figure 2:**
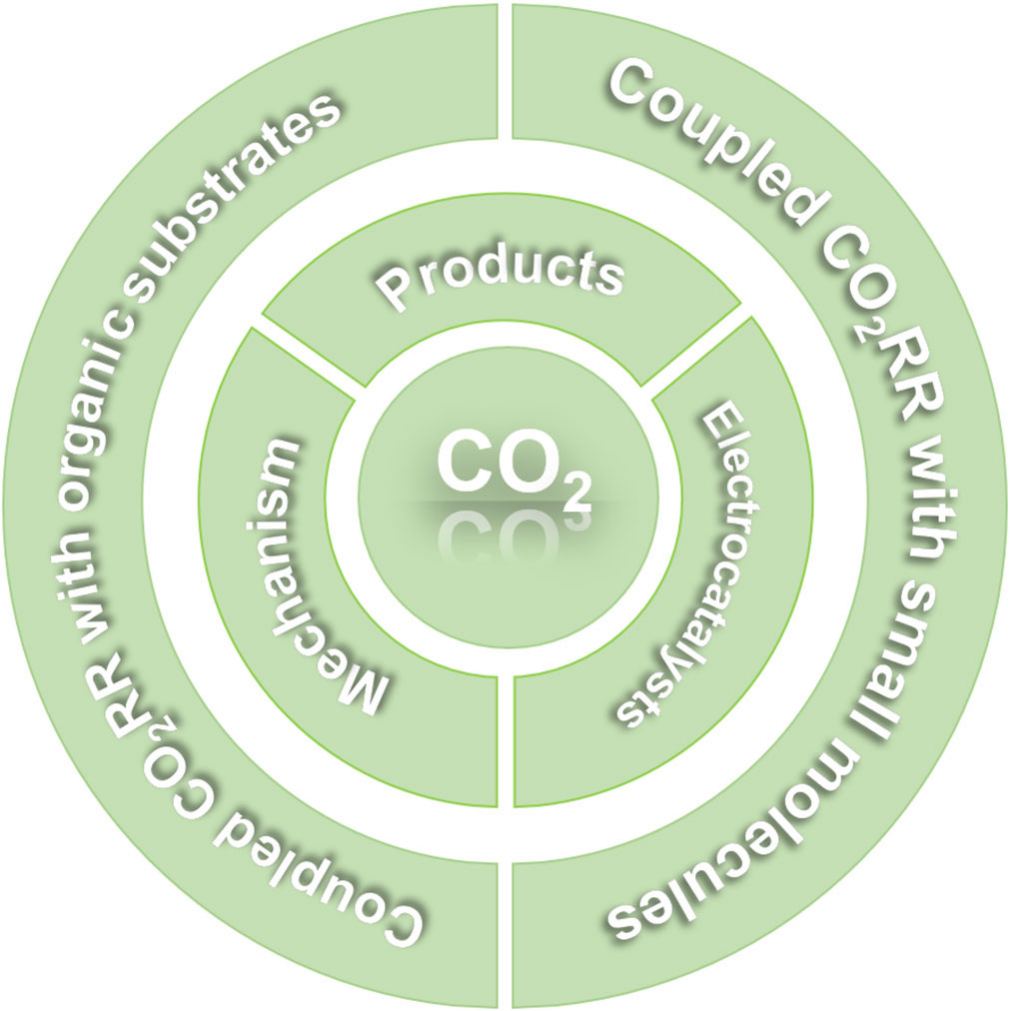
Schematic of the key aspects in the reduction of CO_2_.

In this Review, we focus our discussion on the mechanism and development of CO_2_RR and the coupled CO_2_RR with small molecules and organic substrates (**Figure** **2**). We begin with an introduction to the reaction mechanisms of direct CO_2_RR and present the development of various products separately, followed by the development of electrocatalysts. We then present the principles and methodologies involved in the coupled CO_2_RR with small molecules and organic substrates. We also discuss that some developments in CO_2_RR can be used for reference in coupled CO_2_RR. Several latest developments are specifically introduced. Finally, we conclude the challenges and perspectives on the applications of the coupled CO_2_RR. Overall, reactions based on CO_2_ reduction can provide a more valuable solution to the greenhouse effect.

## 2 Direct CO_2_RR

CO_2_ is a chemically inert molecule and hard to be activated. In aqueous electrolytes, the activation energy of CO_2_ can be decreased using appropriate electrocatalysts.^[^ ^27^ ^]^ However, HER is the main competing side reaction and reduces the FE of CO_2_RR.^[^ ^28^ ^]^ In addition, the CO_2_RR product diversity and low selectivity also present significant challenges to realize practical industrialization of CO_2_RR. Therefore, researchers should further explore the reaction mechanisms of CO_2_RR and understand how to optimize the electrolytic conditions including electrocatalysts.

### 2.1 Mechanism

**Figure 3:**
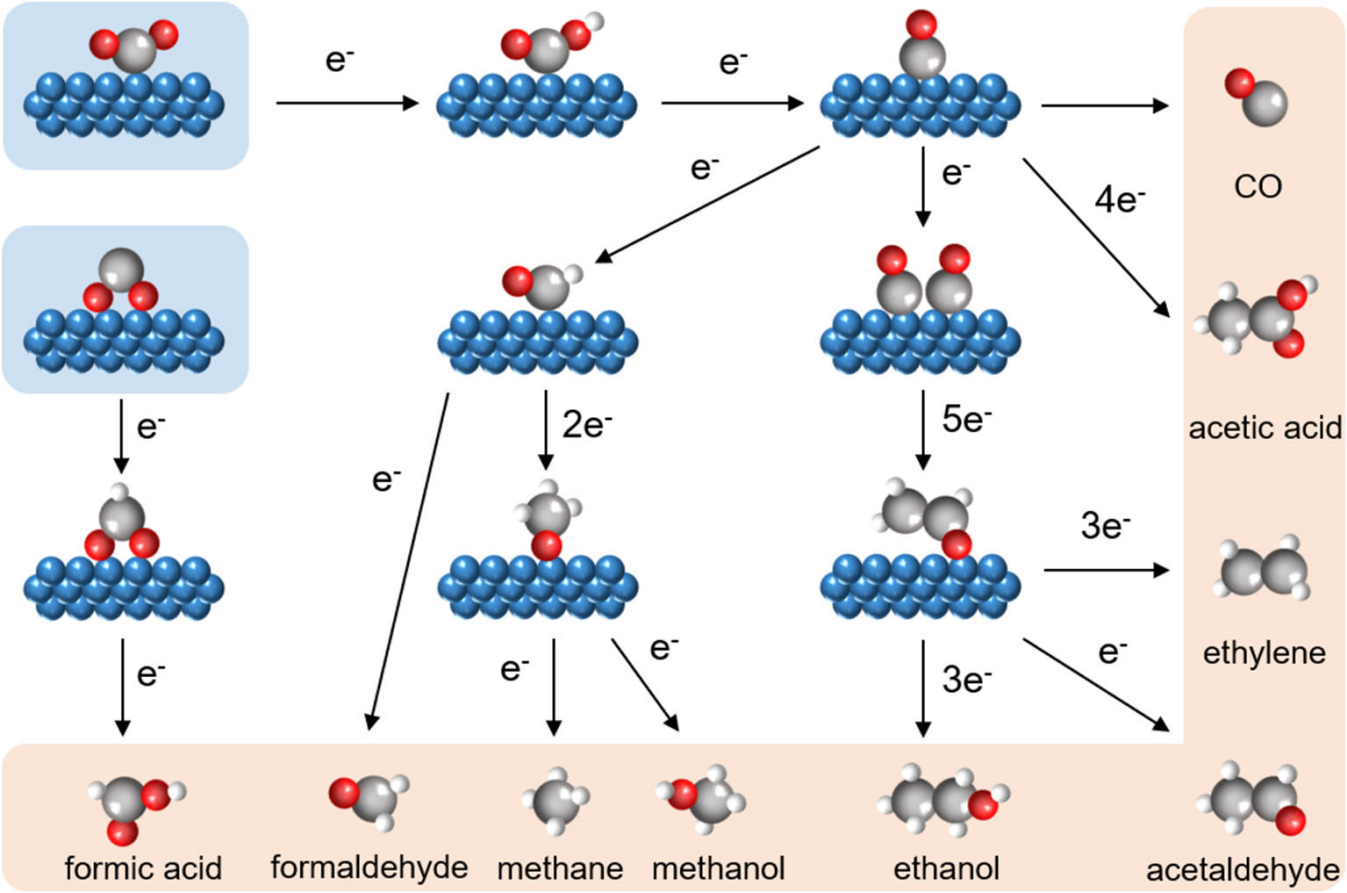
Brief of reaction pathways for CO_2_RR toward different products. Blue spheres, catalysts; grey spheres, carbon; red spheres, oxygen; white spheres, hydrogen.

The reaction pathway of CO_2_RR with H_2_O involves the transfer of multiple electrons and protons, depending on the needs of different products. **Figure** **3** briefly shows the activation routes for some common products in CO_2_RR. The adsorption and activation of CO_2_ is the first step in CO_2_RR, which is generally considered to require a negative redox potential of –1.90 V (vs standard hydrogen electrode, SHE) to transfer one electron to CO_2_.^[^ ^29^ ^]^ With suitable electrocatalysts, bonding with CO_2_ to form a stable CO_2_^•−^ radical can decrease overpotential. There are two primary modes for the adsorption of CO_2_ on the surface of an electrocatalyst, *COO and *OCO, in which through *OCO only formic acid is formed, whereas *COO can lead to many other possible products.^[^ ^30^ ^]^ From theoretical calculations and experiments, researchers have generally agreed that the transition metal materials prefer to bind CO_2_ via carbon (i.e., *COO) and generate CO or *CO intermediate, whereas main group metals like Sn and Bi prefer to bind CO_2_ via oxygen (i.e., *OCO) and produce formic acid.^[^ ^31^ ^]^ CO and formic acid are related to a two‐electron transfer process with only one intermediate, which can be achieved in a relatively high current density and selectivity at low overpotentials. Generally, molecule reactants prefer nucleophilic attack on CO_2_ from carbon site, so that the *OCO binding mode on catalysts may be enhanced to promote coupled CO_2_RR, and main group metals can play a greater role.

For higher energy density and industrial value, C_2_ products such as ethanol and ethylene have attracted substantial attention. To further reduce *CO into multicarbon products, it involves the formation of *CO dimerization and many possible arrangements of the protonation sites.^[^ ^32^ ^]^ The copper‐based catalysts, which are capable of *CO dimerization, also suffer from low product selectivity.^[^ ^33^ ^]^ The product selectivity usually depends on which sites CO_2_ binds to. When CO_2_ is adsorbed on Cu(100), C_2_ products tend to form, whereas on Cu(111), both methane and ethylene can be the primary products.^[^ ^34^ ^]^ The multicarbon products such as ethanol, acetic acid, and ethylene are related to more electron and proton transfer than C_1_ products, and thus the reaction rate and selectivity are hard to achieve toward practical applications.^[^ ^2^ ^]^ For instance, ethylene production involves the transfer of 12 electrons, whereas CO involves 2. Therefore, it is necessary to filtrate and optimize catalysts to make them capable of stronger charge transfer ability. Higher‐order (C_3+_) hydrocarbon products can also be generated on copper‐based catalysts.^[^ ^35^ ^]^ Nevertheless, the selectivity is lower than 20% and a more detailed mechanism understanding is still insufficient. As CO_2_RR involves multiple reaction steps and intermediates, molecule reactants may react with different CO_2_RR intermediates at any step, which substantially increases the product diversity. Therefore, by tuning the adsorption and desorption capacity of electrocatalysts to different reaction intermediates, target products can be selectively obtained from coupled CO_2_RR.

### 2.2 Allocation of Products

Catalysts have specific selectivity for different products. The formation of C_1_ products (e.g., CO, methane, methanol, and formic acid) can occur in many materials, whereas C_2_ products (e.g. ethanol, ethylene, and acetic acid) have been mainly reported on copper‐based catalysts.^[^ ^4^ ^]^

CO is one of the most common products in current studies. The reduction of CO_2_ to CO relates to a two‐electron transfer process. CO is produced via an intermediate that CO_2_ binds to catalysts through carbon atom and form a carboxyl intermediate *COOH. Up to date, Au, Ag, and molecular catalysts are excellent catalysts for CO production, and the FE can be almost up 100%.^[^ 36, 37, 38 ^]^ For example, cobalt phthalocyanine (Pc) can convert CO_2_ to CO with a selectivity > 95% at 150 mA·cm^−2^.^[^ ^39^ ^]^ The initial binding of CO_2_ on metal catalysts involves a concerted proton–electron transfer (CPET), whereas on molecular catalysts it involves an electron transfer‐mediated CO_2_ binding step.^[^ ^18^ ^]^ In CPET, CO_2_ is directly converted into *CO without the formation of CO_2_^•−^. The unique electron transfer‐mediated reaction mechanism enables the existence of CO_2_^•−^, which serves as the active center to connect with molecule reactants. It can be seen that the molecular catalysts present great potentials as catalysts for coupled CO_2_RR.

Formic acid is more readily produced when CO_2_ binds to catalysts by one or two oxygen atoms and forms *OCHO intermediate. P‐block main group metals like In, Sn, Pb, and Bi prefer to generate HCOO^−^ or HCOOH.^[^ ^14^ ^]^ In recent years, substantial research has been studying Bi‐based catalysts due to their less toxic and more environmental friendliness. Researchers have found that the Bi‐based catalysts can undergo a structural change during CO_2_RR, construct defect‐rich catalyst sites, and thus optimize the adsorption capacity to *OCHO.^[^ 14, 40 ^]^

*CO can be converted into methanol and methane without the processing of *CO dimerization.^[^ ^41^ ^]^ Methanol, a liquid fuel with high economic value, has been reported by Cu‐based catalysts, molecular catalysts, and single‐atom catalysts.^[^ ^37^ ^]^ Methane is one of the research hotspots in CO_2_RR in recent years, and several researches have reported high FE values by coordination compounds and copper‐based catalysts.^[^ ^42^ ^]^

Ethylene and ethanol are among the most focusing products to date, due to their high energy density and industrial value. The key step to obtain most C_2_ products is the formation of *CO dimerization, which is more likely to occur on the Cu surface. Thus, most of the literature on C_2_ products have used Cu‐based catalysts,^[^ ^43^ ^]^ whereas most reported FE values of ethanol and ethylene on copper electrode are still less than 70% at high current densities. In addition to copper‐based materials, C_2_ products can be produced on nitrogen (N)‐doped carbon,^[^ ^44^ ^]^ NiGa,^[^ ^45^ ^]^ and PdAu^[^ ^46^ ^]^ catalysts, but not as efficiently as copper. For example, an FE value of 77.6% for producing acetate was reported on N‐doped nanodiamond.^[^ ^47^ ^]^ Obtaining further long‐chain products like propanol has still remained as a large challenge and only several works using Cu‐based catalysts have reported the conversion of CO_2_ to propanol.^[^ 48, 49 ^]^

### 2.3 Design of Catalysts

In addition to the numerous products and low selectivity for CO_2_RR, HER also participates in competing with the electrons, so the transformation process needs to be optimized. The CO_2_RR involves the adsorption, activation, and desorption of CO_2_ molecules on a catalyst's surface. Although homogeneous molecular catalysts can present high selectivity, the poor stability, high cost, and excessive postseparation steps inhibit their practical application.^[^ ^39^ ^]^ Major efforts have thus been devoted to the study of heterogeneous catalysts, such as carbon‐based materials, metals (oxides), alloy, and organic molecules. **Table** **1** shows the development of the main types of catalysts for CO_2_RR in recent years, showing their optimization methods and performance achievements. In general, the advances involve the electronic structure optimization, catalysts’ reconstruction, and fabrication engineering, which affect the transporting behavior of electrons and ions on catalysts’ surface. Below we briefly introduce several main catalyst design principles.

**Table 1:** Summary for the main types of CO_2_RR electrocatalysts and their optimal methods

Types	Catalysts	Electrolytes	Activity	Method	Ref.
Cu‐based catalysts	Cu nanocavity	1 m KOH	FE(propanol) = 21%	Reducing Cu_2_O cavities into Cu nanocavity	[35]
	Cu/gas‐diffusion electrode (GDE)	10 m KOH	FE(C_2_H_4_) = 70%	Sputtering Cu on GDE or PTFE substrates and using alkaline electrolyte to construct abrupt reaction interface	[28]
	Ag‐doped Cu	1 m KOH	FE(*n*‐propanol) = 33%	Immersing the Cu‐coated GDE in AgNO_3_ aqueous solution at 65 °C	[81]
	Ce(OH)_*x*_‐doped Cu	1 m KOH	FE(C_2_H_5_OH) = 43%	Electrochemically depositing hydroxides on sputtered Cu/PTFE	[7]
	Fluorine‐modified copper	1 m KOH	FE(C_2‐4_) = 43%	Electroreduing Cu(OH)F in 1 M KOH	[82]
	N‐doped nanodiamond/Cu	0.5 m KHCO_3_	FE(C_2_ oxygenates) = 63%	Depositing nanodiamond on Si wafer and nitriding by microwave‐plasma‐assisted chemical vapour deposition (MPCVD) and sputtering Cu on it	[83]
	Cu/perfluorinated sulfonic acid ionomers	7 m KOH	I(C_2_H_4_) = 1.3 A cm^−2^	Sputtering perfluorinated sulfonic acid ionomers on sputtered Cu	[6]
	Cu/N−C	1 m KOH	FE(C_2_H_5_OH) = 52%	Sputtering 50 nm N−C layer on sputtered Cu	[84]
	Double sulfur vacancy‐rich CuS	1 m KOH	FE(*n*‐propanol) = 15.4%	Using CuS as the cathode for lithium‐ion battery and charge−discharge	[49]
	Cu–polyamine hybrid catalysts	1 m KOH	FE(C_2_H_4_) = 87%	Electrodepositing Cu and polymer on GDE	[17]
Molecular electrolysts	Polyoxometalatemetalloporphyrin organic framework	0.5 m KHCO_3_	FE(CO) = 99%	Integration of [*ε*‐PMo_8_^V^Mo_4_^VI^O_40_Zn_4_] cluster and metalloporphyrin endows PMOF‐oriented electron transmission	[85]
	CoPc–NH_2_/carbon nanotubes (CNT)	0.1 m KHCO_3_	FE(methanol) = 15.4%	Diffusing CoPc−NH_2_ on CNTs	[37]
	NiPc/CNTs	1 m KHCO_3_	FE(CO) = 99.5%	Diffusing NiPc molecules on CNTs	[86]
	Metalloporphyrin‐tetrathiafulvalene‐based COF	0.5 m KHCO_3_	FE(CO) = 91.3%	Tetrathiafulvalene serving as electron donator or carrier and constructing an oriented electron transmission pathway with metalloporphyrin	[87]
Carbon‐based catalysts	Atomically Ni(I)−NG	0.5 m KHCO_3_	FE(CO) = 97%	Constructing monovalent Ni(I) atomic center with a *d* ^9^ electronic configuration	[88]
	Sb SA/NC	0.5 m KHCO_3_	FE(formate) = 94%	Pyrolyzing SbCl_3_, dicyandiamide, and trimesic acid at 800 °C	[89]
	Mn−N_3_−C	0.5 m KHCO_3_	FE(CO) = 98%	Annealing multi‐walled carbon nanotubes (MWCNTs), dicyandiamide, and Mn ions at 873 K for 1 h.	[90]
	Ni single atoms/PCFM	0.5 m KHCO_3_	FE(CO) = 88%	Annealing the spun fibers consist of ZIF‐8 and Ni ions to construct Ni single atoms/PCFM	[91]
Metal catalysts	Pd−B/C	0.1 m KHCO_3_	FE(formate) = 70%	Using dimethylamine borane as reducing agent to prepare B‐doped Pd/C	[92]
	Defect‐rich ultrathin Pd nanosheet	0.1 m KHCO_3_	FE(CO) = 93%	Electrochemically reconstructing (111)‐rich Pd nanosheets into (100)‐rich crumpled Pd nanosheets	[93]
Mesoporous PdAg nanospheres	0.1 m KHCO_3_	FE(formate) = 100%	Co reduction of Pd and Ag precursors in aqueous solution using DOAC as the structure‐directing agent	[94]
Palladium–silver alloy nanowires	0.1 m KHCO_3_	FE(formate) = 95%	Co reduction of Pd and Ag precursors in aqueous solution using DHDAC as the structure‐directing agent	[95]
MOF−AuPd	0.5 m KHCO_3_	FE(HCOOH) = 99%	Confining atomically dispersed Au on tensile‐strained Pd nanoparticles	[96]
Metal oxide/carbide/sulfide catalysts	S−In	0.5 m KHCO_3_	FE(formate) = 85%	Electrochemically reducing S‐containing In_2_O_3_ into S−In	[97]
	β‐Bi_2_O_3_ double‐walled nanotubes	1 m KOH	FE(formate) = 98%	Electrochemically converting Bi_2_O_3_ NTs into Bi with defects	[40]
	Amorphous InO_*x*_ nanoribbons	0.5 m NaHCO_3_	FE(formate) = 91.7%	Calcination treating InO_*x*_ under air or H_2_ to obtain InO_*x*_ with tunable O vacancy	[98]
	Sn(S)	0.1 m KHCO_3_	FE(formate) = 93%	Using atomic layer deposition of SnS_*x*_ followed by a reduction process	[99]

**Figure 4:**
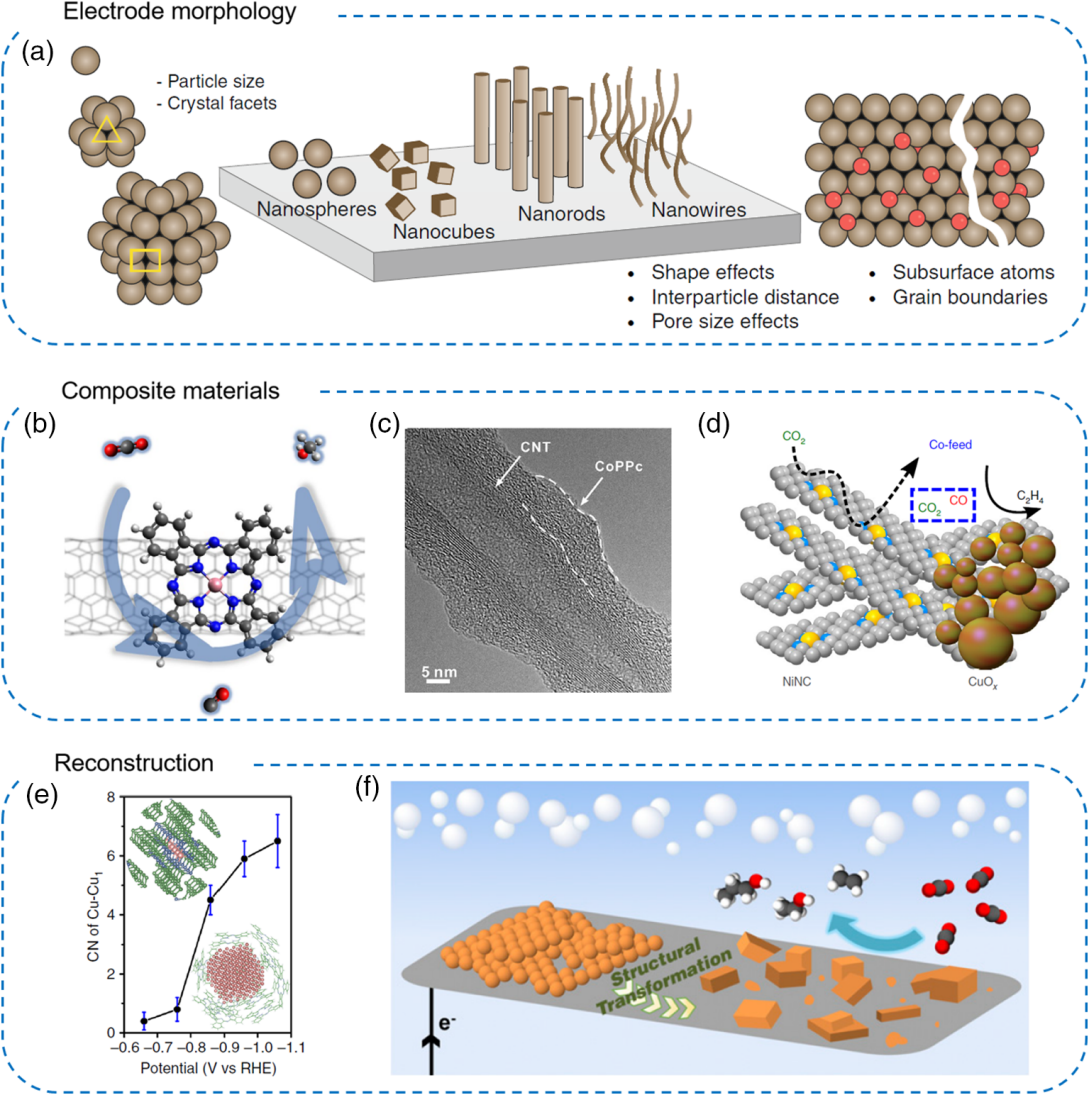
Representative structural design of electrocatalysts. a) Morphology design. Reproduced with permission.^[^ ^18^ ^]^ Copyright 2019, Springer Nature. b) Core−shell structure. Reproduced with permission.^[^ ^37^ ^]^ Copyright 2019, Springer Nature. c) Tandem catalysts. Reproduced with permission.^[^ ^55^ ^]^ Copyright 2017, Elsevier. d) Hybrid catalysts. Reproduced with permission.^[^ ^56^ ^]^ Copyright 2019, Springer Nature. e,f) Catalysts’ reconstruction. Reproduced with permission.^[^ ^57^ ^]^ Copyright 2018, Springer Nature. Reproduced with permission.^[^ ^58^ ^]^ Copyright 2017, National Academy of Sciences.

With the rational design and development of electrocatalysts for CO_2_RR, high activity and selectivity can be obtained, which are attributed to high specific surface area, abundant active sites, local morphology, and/or undercoordinated sites (**Figure** **4**a).^[^ ^18^ ^]^ The microstructures of electrodes have great effects on the electron and mass transfer of reactants, whereas the selectivity of CO_2_RR is sensitive to the local concentration of CO_2_ and intermediates. Thus, optimizing design based on surface structure can be an indispensable development direction of CO_2_RR electrocatalysts.

The pretreatments of catalysts have often been used to enhance the activity of CO_2_RR. The adjustment of appropriate particle sizes and morphologies in nanoscale has been shown to influence the CO_2_RR activity through more undercoordinated sites.^[^ ^50^ ^]^ Exposing more crystal faces can lead to higher selectivity for specific products, like Cu(100) in favor of ethylene.^[^ ^34^ ^]^ Subsurface atoms and grain boundaries have been observed in oxide‐derived (OD) electrodes, which were acquired by reducing metal oxides.^[^ 51, 52 ^]^ A high selectivity toward CO was recorded on OD Au, and obvious improvements in selectivity toward ethanol were also detected on OD Cu. The reasons for the increased activity and selectivity of CO_2_RR on OD electrodes are still being debated. One possible explanation is that the CO_2_^•−^ can be stabilized by the presence of subsurface oxygen atoms or grain boundaries.^[^ 53, 54 ^]^ Constructing composite materials is another practical approach. Cobalt phthalocyanine is known as a catalyst with high CO selectivity. Wang and coworkers immobilized cobalt phthalocyanine on carbon nanotubes and realized the six‐electron reduction of CO_2_ to methanol with FE higher than 40% via a distinct domino process (Figure 4b).^[^ ^37^ ^]^ Interestingly, for the polymeric form of phthalocyanines supported on carbon nanotubes, a high FE of 90% for converting CO_2_ to CO was achieved (Figure 4c).^[^ ^55^ ^]^ It is worth investigating the influence of the present form of metal phthalocyanines on selectivity. As the evolution of C_2+_ products involves C–C coupling and mass migration, it is reasonable to design tandem catalysts to achieve the migration and further reduction of intermediates. Strasser et al. reported a tandem catalyst consisting of CuO_*x*_ nanoparticles and Ni–N–C, which is efficient to reduce CO_2_ to CO (Figure 4d).^[^ ^56^ ^]^ The prepared catalyst showed a significant improvement of catalytic C_2_H_4_ performance by enhancing the cross‐coupled CO_2_–CO reactive pathway.

Due to the highly negative applied potentials during CO_2_RR, most metallic compounds can be reduced to zero‐valent metals. In recent years, the reconstruction process of catalysts in CO_2_RR has been emerging as one of the research hotspots. Wang and coworkers found that the Cu‐based metal organic framework (MOF), molecular catalysts, and phthalocyanine underwent structural reconstruction to metallic Cu clusters under CO_2_RR working potentials (Figure 4e).^[^ ^57^ ^]^ Among them, CuPc was reversibly transformed to Cu clusters with a size of ≈2 nm with released Pc molecules near Cu clusters, resulting more undercoordinated sites that favored CH_4_. Yang and coworkers reported that uniform Cu nanoparticles anchored on carbon paper underwent structural transformation during CO_2_RR into scattered cube‐like particles and showed improved C_2+_ products selectivity (Figure 4f).^[^ ^58^ ^]^ The design and optimization of electrocatalysts for CO_2_RR involve the influence on the adsorption capacity of reactants, which can provide valuable catalyst suggestions for CO_2_RR with small molecules and organic substrates.

## 3 Coupled CO_2_RR with Small Molecules

The conversion of CO_2_ and water into valuable chemical products represents a reliable pathway to solve greenhouse effect, but the product is limited in carbon hydroxide when pure CO_2_ is used as the reactant. The combination of CO_2_ and other heteroatoms, such as N, S, and Br from small‐molecule reactants, can produce higher valuable products.^[^ ^22^ ^]^ For example, combining CO_2_ with Br elements can lead to the product of bromohydrins and 2‐bromoethanol, whereas combining CO_2_ with N elements may lead to *N*‐ethylacetamide, *N,N*‐dimethylacetamide, and so on.^[^ 24, 59 ^]^

**Figure 5:**
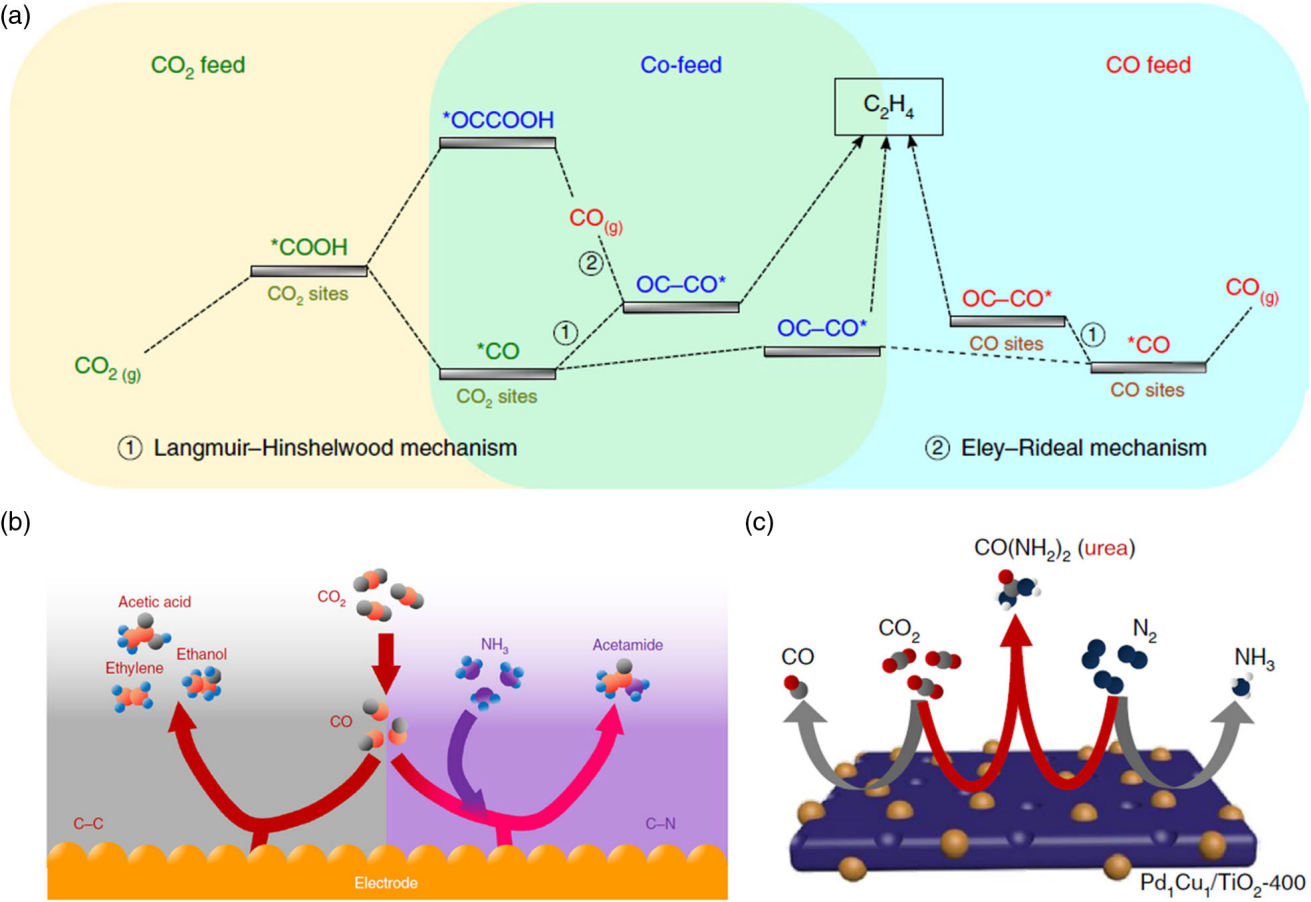
Coupled CO_2_RR with small molecules. a) Mechanism diagram of different gas feeds for CO_2_RR. Reproduced with permission.^[^ ^56^ ^]^ Copyright 2019, Springer Nature. b) A schematic that depicts CO electrolysis with NH_3_ to produce acetamide. Reproduced with permission.^[^ ^24^ ^]^ Copyright 2019, Springer Nature. c) Schematic diagram for urea synthesis from CO_2_ electrolysis with N_2_. Reproduced with permission.^[^ ^62^ ^]^ Copyright 2020, Springer Nature.

As aforementioned, the formation of *OCCO dimerization intermediate is the limited step toward C_2+_ products, in which the key factors include the adsorption capability of catalysts to CO and local concentrations of CO or *CO. To increase the proportion of *OCCO dimerization, Wang and coworkers introduced mixed CO_2_/CO feeds to enhance the *CO surface coverage (**Figure** **5**a).^[^ ^56^ ^]^ By isotope‐labeled (^12^C/^13^C) and operando differential electrochemical mass spectrometry (DEMS), a *CO (from CO)–*CO (from CO_2_) cross‐coupling pathway was proposed, suggesting that the additional CO did not compete with CO_2_ for active sites but served for synergistic catalysis. With cofed CO and *OCCO coverage, the production of C_2_H_4_ was enhanced over 50%. The ketene intermediate (*C═C═O) formed from the subsequent protonation of *OCCO dimerization is known to be highly reactive with nucleophilic species. For example, with the nucleophilic attack of OH^−^, an enhanced acetate selectivity was achieved in CO electroreduction.^[^ ^60^ ^]^ Taking advantage of this feature, introducing specific nucleophilic species into CO_2_/CO electrolysis system can result in a wide variety of valuable products. Jouny et al. reported that cooperating with nucleophilic NH_3_ on CO electroreduction, C—N bond was formed from the attack of NH_3_ to ketene intermediate, and nearly 40% FE of acetamide was obtained (Figure 5b).^[^ ^24^ ^]^ By replacing NH_3_ with amino‐containing organics, the concept was successfully extended to the synthesis of *N*‐ethylacetamide, *N*‐methylacetamide, *N,N*‐dimethylacetamide, aceturic acid, and acetic monoethanolamide, which can be used as precursors to construct larger molecules. In addition, with the introduction of NH_3_ or amino‐containing organics, the formation of C_2+_ products was suppressed, indicating that the attack of nucleophilic species and the subsequent reduction of ketene were competing reactions.

Furthermore, the fixation of N_2_ to NH_3_ has been dominated by the Haber−Bosch process, which accounts for about 2% of the energy consumed in the world.^[^ ^61^ ^]^ It can be more economic and environmental friendly to convert N_2_ into reactive intermediates by electrochemical methods and then coupling with CO_2_RR. Chen et al. reported direct electrochemical coupling of N_2_ and CO_2_ toward urea on PdCu alloy nanoparticles anchored on TiO_2_ nanosheets (Figure 5c).^[^ ^62^ ^]^ When *CO was formed, *N_2_ showed a strong effect on *CO and then formed a urea precursor *NCON*. Due to high formation energy of *NNH, the pathway to NH_3_ formation was inhibited, resulting in a high urea selectivity of 8.92% FE and a urea formation rate of 3.36 mmol g^−1^ h^−1^. Although the productivity of the electrochemical method is still much lower than that of the industrial Haber−Bosch process, it provides a potential pathway to active inert N_2_ using renewable energy. Introducing other molecular reactants can result in product diversity, and *CO plays an important role in binding with molecular reactants. However, up to now, only Cu‐based electrocatalysts have exhibited both high CO_2_ activation capacity and high CO adsorption capacity. Consequently, Cu‐based materials have been the main types of electrocatalysts to activate the coupled CO_2_RR with small‐molecular reactants. Because the coupled CO_2_RR process also depends on the adsorption and activation capacity of catalysts to reactants, the surface structure of morphology, component, and coordination also has great effects on the catalytic performances.

**Figure 6:**
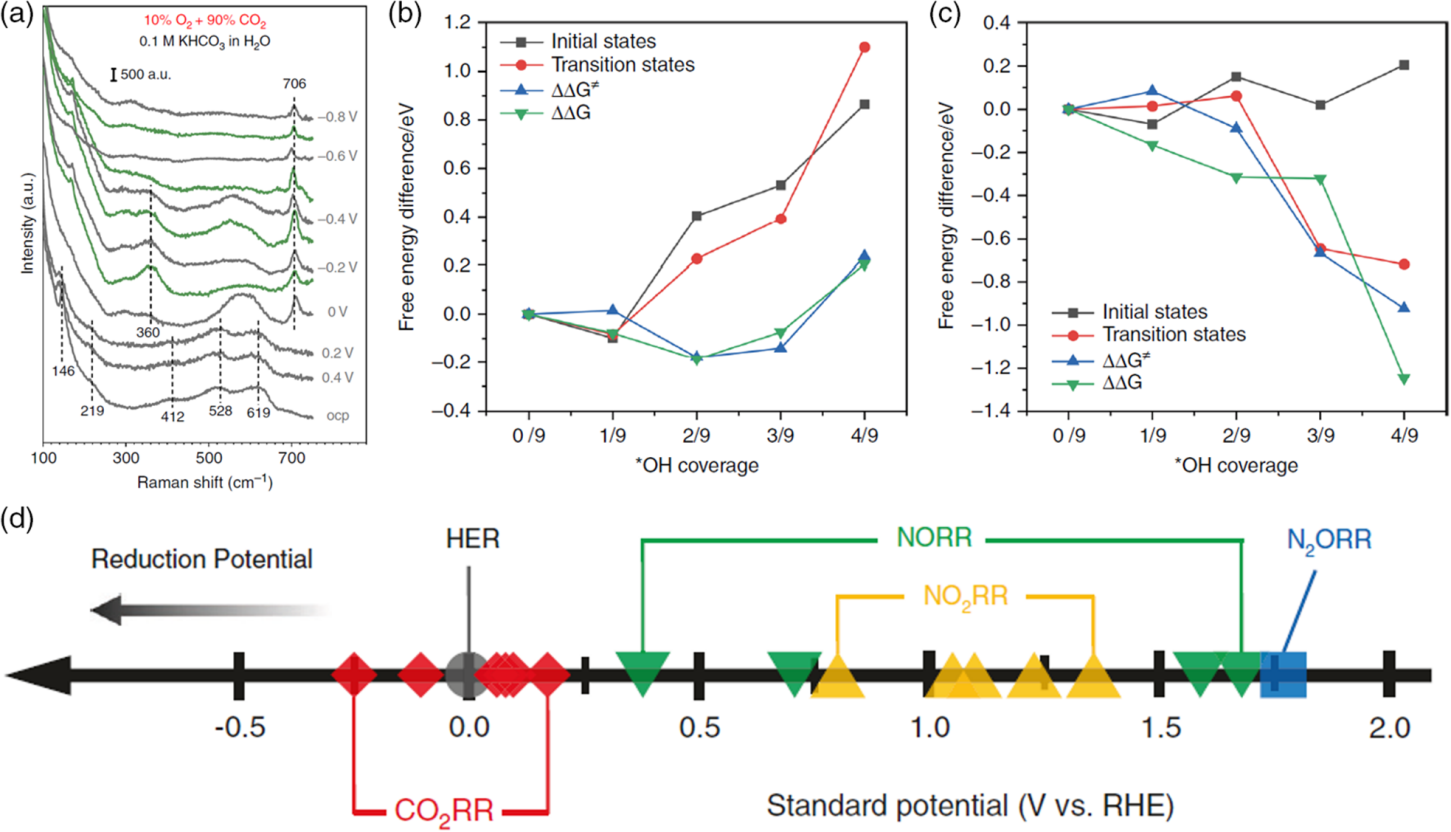
Coupled CO_2_RR with small molecules. a) Raman spectra of Cu catalysts at 90% CO_2_ + 10% O_2_ in 0.1 M KHCO_3_/H_2_O. Free energy diagram of b) *CO dimerization and c) *CO hydrogenation. a–c) Reproduced with permission.^[^ ^63^ ^]^ Copyright 2020, Springer Nature. d) Standard potential versus RHE for CO_2_RR, HER, and NO_*x*_RR. Reproduced with permission.^[^ ^65^ ^]^ Copyright 2020, Springer Nature.

In addition to extra reactants introduced, CO_2_ reactant can also be mixed with other gases. Bringing low concentrations of O_2_ in CO_2_ feeds can also affect the behavior of CO_2_RR. The surface hydroxyl species formed by oxygen reduction reduce the formation energy barrier of oxygenates and hydrocarbon products and improve the production rate up to 216‐fold.^[^ ^63^ ^]^ In situ surface‐enhanced Raman spectroscopy (SERS) was adapted to detect the surface species on Cu microparticles. In pure Ar and CO_2_ atmospheres, only the disappearance of surface Cu_2_O signals was detected as the potential became more negative. With the introduction of O_2_, a distinct band at 706 cm^−1^ appeared, which was attributed to the surface hydroxyl species (**Figure** **6**a). Theoretical calculations indicated that as the *OH coverage increased, the initial state of *CO dimerization became unstable and led to the decrease in reaction barrier and subsequently enhanced C_2+_ product formation (Figure 6b). Moreover, the additional hydrogen bonds formed between *CHO and *OH resulted in the stabilization of *CHO intermediate and improved the hydrocarbons’ product selectivity (Figure 6c). Indeed, CO_2_ produced from industrial processes inevitably contains a small amount of CO, NO_*x*_, SO_2_, and other impurities. Some of these impurities can promote CO_2_RR or coelectrolysis, whereas others have a negative effect. For instance, due to more positive reduction potentials of NO_*x*_, the NO_*x*_ reduction reaction can be dominant and reduce the FE of CO_2_RR (Figure 6d). Jiao and coworkers found that the gaseous impurities such as NO_*x*_ and SO_2_ with more positive reduction potentials led to the products’ FE loss in CO_2_RR, especially for C_2+_ products on Cu‐based electrocatalysts.^[^ 64, 65 ^]^ Even trace (up to 1%) impurities can cause significant negative benefits. With the demand of more electrons involved in NO and NO_2_ reduction, the weakening on CO_2_RR was more severe. Interestingly, the gaseous impurities did not cause permanent damage on electrocatalysts under the introduction of NO_*x*_. Once pure CO_2_ was supplied, the similar CO_2_RR performance was recovered. However, by incorporating CO_2_ with 1% SO_2_, the SO_2_ impurity contaminated the Cu catalysts and caused the formation of Cu_2_S and thus shifted the selectivity from C_2+_ products toward formate. Thus, impure reactants of O_2_, CO, and N_2_ can be used in cocatalysis, whereas NO_*x*_ and SO_2_ may reduce the efficiency of CO_2_RR as the reduction of NO_*x*_ and SO_2_ is thermodynamically more favorable.

Until now, there are relatively few studies on coupled CO_2_RR with small molecules, and most are still preliminary demonstration of the feasibility and reaction mechanism. As CO_2_RR involves dozens of intermediates, the small‐molecular reactants may bind different intermediates and result in complicated mixtures of products. In addition, the inhibiting/promoting effects of additional molecular reactants have to be further probed. Thus, the unclear reaction mechanism hinders further industrial exploration of higher reactivities. Resolving these problems is of great significance to the realization of using CO_2_ feedstock from air or fossil‐burning sources, due to the high cost of direct air‐capture technologies and CO_2_ separation.

## 4 Coupled CO_2_RR with Organic Substrates

To obtain long‐chain product with high energy density, it can cost dozens of electrons from direct CO_2_RR, for example, from six electrons in producing methanol to 48 electrons in producing octanol. In addition, the product selectivity of C_3_ can only reach less than 20% at present, and the long‐chain product is even lower. The low product selectivity causes low energy efficiency and excessive consumption of electricity, and the separation of products is also difficult and expensive. Thus, the coupled CO_2_RR with organic substrates may become a promising pathway for CO_2_ fixation and construct long‐chain products. CO_2_ has been used as C_1_ synthon in organic synthesis due to its availability, nontoxicity, and recyclability.^[^ ^66^ ^]^ There are several industrialized organic synthesis routes starting from CO_2_.^[^ ^67^ ^]^ For instance, the synthesis of urea involves the reaction of CO_2_ and NH_3_ under high pressures, and the reaction of CO_2_ and epoxides yields alkylene carbonates. There is another kind of reaction called electrocarboxylation reaction, which involves CO_2_ and organic substrates intrigued by electricity to produce carboxylic acids. The carboxylates can be used as important precursors or intermediates for polymers and pharmaceuticals synthesis.^[^ ^25^ ^]^

There are two reaction pathways for electrocarboxylation reaction.^[^ ^68^ ^]^ One is that the reaction starts with the valorization of CO_2_ to CO_2_ radicals (CO_2_^•−^), followed by the reaction with organic molecules. The other one is that the reaction is initiated by the generation of carbanions, followed by its capture of CO_2_ molecules. The mechanism of a certain electrocarboxylation reaction depends on the reduction potentials of organic molecules and CO_2_ molecules. The cathodic reaction of electrocarboxylation generates carboxylic anions and thus needs an anodic reaction that yields cations. The reaction configuration of electrocarboxylation often uses metallic Mg or Al as anode, which generates metallic cations to solidify carboxylic anions from the cathode.^[^ ^11^ ^]^

When it comes to the electrocarboxylation reaction, the staring organic substrates fall into four categories, including CO_2_ reacting with organic substrates containing carbon−carbon double bond (olefins) or carbon−carbon triple bond (alkynes), carbon−oxygen bond (carbonyl compounds), carbon−nitrogen bond (imines), and C—X bond (organic halides, X = Cl, Br, I).

### 4.1 The Electrocarboxylation Reaction of Olefins or Alkynes

**Figure 7:**
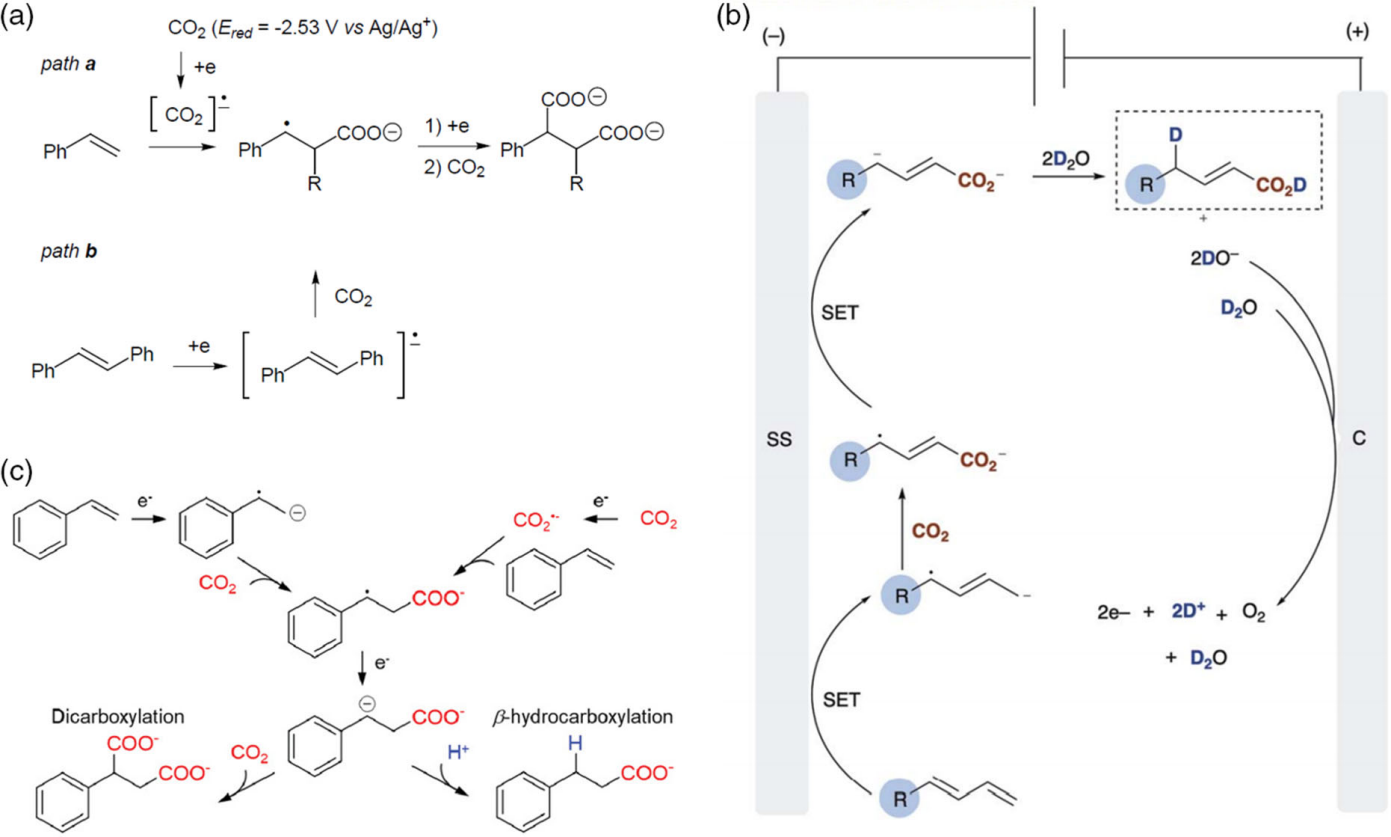
Reactive electrocarboxylation of olefins or alkynes. a) Two reaction pathways for the electrocarboxylation of styrene. Reproduced with permission.^[^ ^69^ ^]^ Copyright 2001, Georg Thieme Verlag. b) Postulated reaction mechanism of electrocarboxylation of dienes. Reproduced with permission.^[^ ^73^ ^]^ Copyright 2020, The Royal Society of Chemical. c) Reaction mechanism of electrochemical β‐selective hydrocarboxylation of styrene. Reproduced with permission.^[^ ^70^ ^]^ Copyright 2019, Wiley.

The electrocarboxylation of olefins or alkynes yields corresponding monocarboxylates or dicarboxylates. Taking styrene as an example, the product distribution, selectivity, and the reaction pathway depend on several factors. There are two reaction pathways for the electrocarboxylation of styrene (**Figure** **7**a), which depends on the reduction potentials of CO_2_ and styrene. When styrene molecules are substituted with electron‐neutral and ‐donating groups, the reduction potential of CO_2_ is relatively lower than the olefin, so the reaction starts with the generation of CO_2_ radical anions.^[^ ^69^ ^]^ The activation of CO_2_ and olefin can also occur simultaneously, as reported in other works.^[^ 70, 71, 72 ^]^ In general, the reduction of alkenes and olefins is the preferred approach. Buckley and coworkers reported an electrosynthetic approach using stainless steel as cathode and triethanolamine (TEOA) and H_2_O as proton sources and achieved a high selectivity of carboxylating diene at *α*‐carbon (Figure 7b).^[^ ^73^ ^]^ Furthermore, it was found that the reduction of diene occurred in preference to that of CO_2_ with higher electron density of the aryl ring. The product selectivity of the electrocarboxylation of styrene can be influenced by the proton sources from the electrolytes (Figure 7c). Nam and coworkers found that when adding a certain amount of water or other proton additives (such as acetic acid) in the anhydrous electrolyte, β‐hydrocarboxylate was produced,^[^ ^70^ ^]^ and the selectivity between dicarboxylates and β‐hydrocarboxylate also depended on the ratio of proton sources to styrene. The side reaction in electrocarboxylation is hard to avoid on the alkenes’/olefins’ preferred pathway, and thus it is challenging to achieve the targeted products with high selectivity. One possible strategy is to use a catalyst that can preferentially reduce CO_2_ with high CO or formate selectivity, as it involves high *COO and *OCO coverage. The alkenes and olefins can bind with CO_2_^•−^ and lead to higher selectivity of carboxylation products.

### 4.2 The Electrocarboxylation Reaction of Carbonyl Compounds

The electrocarboxylation of carbonyl compounds yields α‐hydroxyacids, which can be used as precursors to synthesize anti‐inflammatory agents.^[^ ^74^ ^]^ As carbonyl compounds have the capability of accepting electrons easier than CO_2_, the electrocarboxylation process is more inclined to proceed in the way of preferential reduction of carbonyl compounds. It can be seen from cyclic voltammetry that the electrocarboxylation of acetophenone starts from the generation of acetophenone radical anions, followed by a fast reaction with CO_2_ molecule.^[^ ^75^ ^]^ Similar to the electrocarboxylation reaction of olefins or alkynes, the side reaction also hinders the high selectivity and FEs of targeted products. The byproducts of such a reaction is the dimer of acetophenone radical anions and the corresponding alcohol, which are the hydrate products of acetophenone radical anions.^[^ ^76^ ^]^ Researchers also found that the proton availability of the electrolyte rendered a strong impact on the product selectivity.

### 4.3 The Electrocarboxylation Reaction of Imines

**Figure 8:**
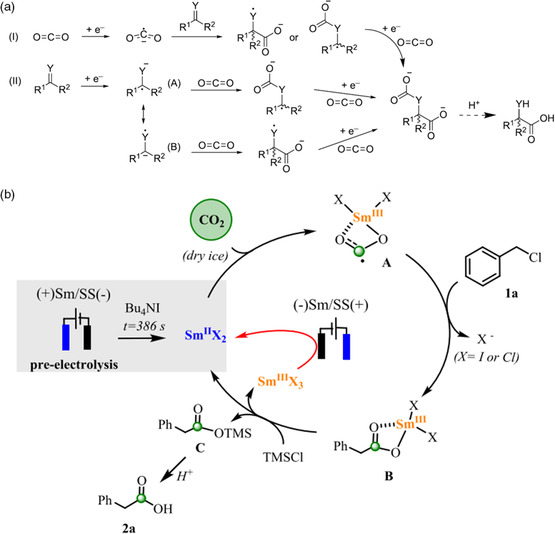
Reactive electrocarboxylation of imines and organic halides. a) Electrocarboxylation mechanism for imines (Y = NH). Reproduced with permission.^[^ ^25^ ^]^ Copyright 2014, Beilstein‐Institut. b) The proposed mechanism of reduction of benzyl halides using samarium sacrificial anode. Reproduced with permission.^[^ ^78^ ^]^ Copyright 2019, American Chemical Society.

The electrochemical carboxylation reaction of imines is capable of yielding non‐natural amino acids. As the case for olefins and alkynes, there are also two reaction pathways existing in the electrocarboxylation of imine compounds, starting with the reduction of CO_2_ or organic substrates (**Figure** **8**a).^[^ ^25^ ^]^ Nevertheless, the pathway II is dominant, in which the imine compound substrates are reduced first, and CO_2_ is fixed on C or N atom depending on the electronegativity of substituent groups. After binding a second CO_2_, a carboxylate intermediate is formed and then converted to hydroxy acid or amino acid after acid hydrolysis.

### 4.4 The Electrocarboxylation Reaction of Organic Halides

The halide atoms of organic halides can also be substituted by carboxyl through the electrochemical carboxylation reaction. The electrocarboxylation of organic halides is initiated by the generation of reactive halide anions, which can be reduced by another electron with the engagement of CO_2_ yielding a monocarboxylate anion.^[^ ^25^ ^]^ Many works on the electrocarboxylation of organic halides use Ag as the cathode catalyst.^[^ ^77^ ^]^ Gennaro et al. reported using Ag cathode to decrease the reduction potentials of benzyl chlorides with 0.6 V than using Hg as cathode catalyst.^[^ ^77^ ^]^ Compared with Hg and glass carbon electrodes, the reduction of benzyl chlorides at Ag electrode related to the interaction of benzyl chloride and its intermediates with Ag, and the decrease in overpotential was attributed to the kinetics promotion effect. Sacrificial anode method is also a useful means to activate CO_2_ and organic halides.^[^ 78, 79 ^]^ Mellah and coworkers reported carboxylation of benzyl halides to phenylacetic acids using samarium sacrificial anode (Figure 8b).^[^ ^78^ ^]^ The Sm(II) units produced by the precatalysis process bound with CO_2_ to form Sm^III^—O bonds. After introducing benzyl chloride and oxophilic reagent, the Sm^III^—O bonds were spilt and Sm (III) species were reduced on cathode to regenerate Sm (II) units, releasing the targeted products.

**Figure 9:**
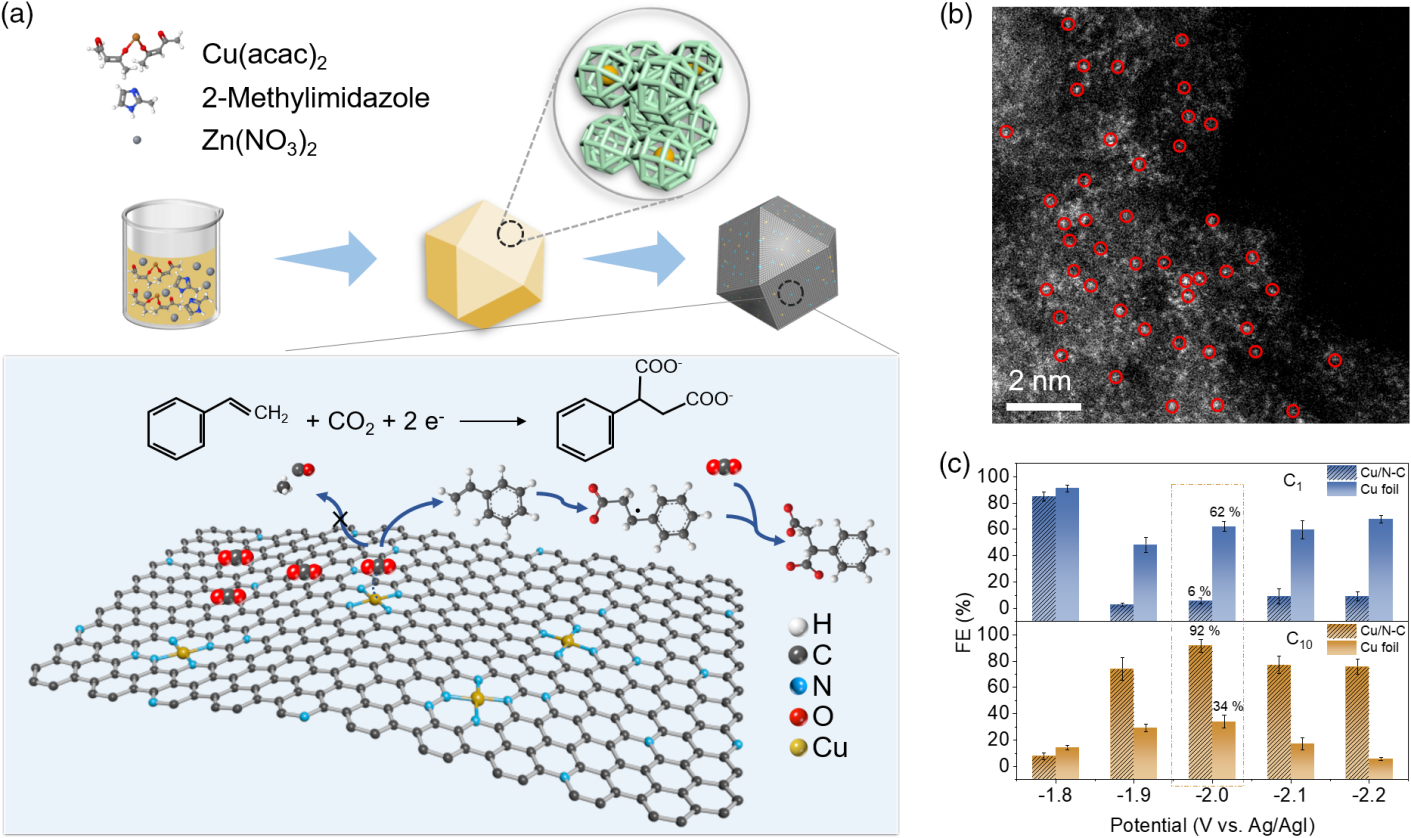
Electrocarboxylation of styrene with CO_2_ using Cu/N−C catalysts. a) Schematic of the procedure to synthesize Cu/N−C catalyst and Cu/N−C favors the carboxylation of styrene over the reduction of CO_2_ to CH_4_ and CO. b) High‐angle annular dark‐field scanning transmission electron microscopy (HAADF‐STEM) energy dispersive spectroscopy (EDS) image of Cu/N−C. c) FE_C1_ and FE_C2+_ on Cu/N−C and Cu foil at different working potentials. Reproduced with permission.^[^ ^80^ ^]^ Copyright 2021, Elsevier.

As the electrocarboxylation reaction occurs on the catalysts’ surface and involves the adsorption and desorption of reactants and intermediates on catalysts, rational design of catalysts enables significant improvement in activity, selectivity, and energy efficiency. Noble metal catalysts like Pt, Ag, and Pd have shown great promises on activating carboxylation reaction due to their adequate adsorption energies to organic substrates. However, the scarcity and high cost of noble metals hamper the processing of large‐scale utilization. In addition, the commonly used electrodes are metal rods, wires, or plates and stainless steel, whereas their low surface areas and low activities result in limited performances. Porous electrodes can exhibit the potentials to overcome the rampart. Recently, we reported the electrocarboxylation of styrene with CO_2_ using nitrogen‐coordinated single‐atomic Cu sites on carbon framework (Cu/N−C) catalysts (**Figure** **9**a–c).^[^ ^80^ ^]^ The porous structure of the carbon framework derived from the carbonization of MOF allowed high exposure of active atomic sites and enhanced the carboxylation reaction activity, and the electron‐rich Cu center enabled to adsorb and activate CO_2_ than organic substrates. The FE of electrocarboxylation was up to 92%. Thus, constructing single‐atomic metal catalysts with porous structures can be a promising strategy to optimize reaction activity, product selectivity, and energy efficiency.

## 5 Conclusion and Outlook

In this Review, we have first briefly discussed the current developments of direct CO_2_RR and then focused on the coupled CO_2_RR with small molecules and organic substrates. Substantial progresses in CO_2_RR have been made with the exploration of reaction mechanisms and the designs of electrocatalysts, electrolytes, and electrolyzers. The current researches on the coupled CO_2_RR with small molecules and organic substrates are still in an early and developing stage and mainly focus on the optimization of reaction conditions. Due to their higher economic potentials and impacts, these coupling reactions will be able to attract more attention from researchers. It is essential to develop efficient catalytic systems and understand the entire process from both reaction mechanisms and electrocatalyst functions. The development of CO_2_RR with H_2_O in these areas can serve as an excellent reference for the coupled CO_2_RR. In this regard, we believe that the following aspects can be explored in the near future.

1) *Investigating the correlation between catalyst structures and products.* As mentioned earlier, in CO_2_RR, there is a clear correlation between catalysts and product types, such as Au and Ag, to produce CO, Sn, and Bi to produce formic acid. In this regard, it is important to establish a congruent correlation on the coupled CO_2_RR by extensive experiments combined with machine learning, which can be helpful for improving the study efficiency.

2) *Multicomposition of reactants*. As shown in Sections 3 and 4, the composition of reactants plays a vital role in CO_2_RR. The introduction of a reactant that is beneficial or harmful to a specific reaction may have a significant effect on regulating product selectivity. In this Review, we have only discussed the effects of two components. Incorporating more components may lead to more unexpected performances. This is worthy of in‐depth study by researchers.

3) *Improving the efficiency and utilization of electrocatalysts*. To date, the electrocatalysts used in the coupled CO_2_RR are mainly metal plates and nanoparticles. Recently, single‐site electrocatalysts have been widely reported in CO_2_RR to achieve the highest utilization of active sites. It will be interesting to discover the capability and efficiency for CO_2_^•−^ and *CO reacting with small molecules and organic substrates. In addition, constructing tandem electrocatalysts is also a powerful means to improve the product selectivity. Actually, the catalyst can gradually change during the reaction, and reconstruction always occurs on the surface or bulk of catalysts. Thus, more attention should be paid to the reconstruction process of catalysts and chase down the actual active sites.

4) *Understanding the effects of electrolytes*. In CO_2_RR, the effects of cations, anions, local electric field, and pH have been widely discussed. Whether similar effects also exist in the coupled CO_2_RR is worthy of further exploring. Moreover, electrocarboxylation mainly proceeds in organic electrolytes without H_2_O, whereas the presence of trace water may bring a substantially different behavior of CO_2_RR. Understanding of the effects of electrolytes in the coupled CO_2_RR is important for the development high‐efficient electrolysis system.

5) *Developing new electrolyzers*. The design of electrolyzers has a great influence on the behavior of CO_2_RR. High current density and selectivity can be obtained in flow cells and membrane electrode assembly (MEA) electrolyzers, whereas membrane‐less single cells can facilitate the coupling of cathode and anode products. Furthermore, a series of electrochemical cells may be an additional booster to realize industry applications, in which the reactive intermediates are released from one cell and then transfer to the next cell for further reactions. It is anticipated that the design of electrolyzers will bring in rapid development of the coupled CO_2_RR.

6) *Exploring the synergistic effect of electrocatalysts, electrolytes, and electrolyzers.* The industrial applications of CO_2_RR and coupled CO_2_RR involve all aspects including electrocatalysts, electrolytes, electrolyzers, and so on. For example, by depositing Cu nanoparticles on a polytetrafluoroethylene (PTFE) gas diffusion electrode, choosing high‐concentration alkaline electrolytes to avoid HER, and assembling flow cells, the onset potential of ethylene evolution was achieved at –0.165 V versus reversible hydrogen electrode (RHE) with 70% of FE in 10 m KOH electrolytes.^[^ ^28^ ^]^

There are still many challenges and unknowns that have to be solved for the development of the coupled CO_2_RR. The concepts and design rules in CO_2_RR about reaction mechanisms and electrocatalysts can serve as useful references for promoting the coupled CO_2_RR. Through the optimization of all aspects from the reaction mechanisms to electrolyzers, it is expected that the coupled CO_2_RR can make revolutionary breakthroughs in the near future.

## Conflict of Interest

The authors declare no conflict of interest.
